# Induction of Eosinophil Apoptosis by the Cyclin-Dependent Kinase Inhibitor AT7519 Promotes the Resolution of Eosinophil-Dominant Allergic Inflammation

**DOI:** 10.1371/journal.pone.0025683

**Published:** 2011-09-30

**Authors:** Ana L. Alessandri, Rodger Duffin, Andrew E. Leitch, Christopher D. Lucas, Tara A. Sheldrake, David A. Dorward, Nik Hirani, Vanessa Pinho, Lirlândia Pires de Sousa, Mauro M. Teixeira, John F. Lyons, Christopher Haslett, Adriano G. Rossi

**Affiliations:** 1 Medical Research Council Centre for Inflammation Research, The Queen's Medical Research Institute, University of Edinburgh, Edinburgh, Scotland, United Kingdom; 2 Imunofarmacologia, Departamento de Bioquímica e Imunologia, Instituto de Ciências Biológicas, Universidade Federal de Minas Gerais, Belo Horizonte, Brazil; 3 Astex Therapeutics, Cambridge, England, United Kingdom; Fundação Oswaldo Cruz, Brazil

## Abstract

**Background:**

Eosinophils not only defend the body against parasitic infection but are also involved in pathological inflammatory allergic diseases such as asthma, allergic rhinitis and contact dermatitis. Clearance of apoptotic eosinophils by macrophages is a key process responsible for driving the resolution of eosinophilic inflammation and can be defective in allergic diseases. However, enhanced resolution of eosinophilic inflammation by deliberate induction of eosinophil apoptosis using pharmacological agents has not been previously demonstrated. Here we investigated the effect of a novel cyclin-dependent kinase inhibitor drug, AT7519, on human and mouse eosinophil apoptosis and examined whether it could enhance the resolution of a murine model of eosinophil-dominant inflammation *in vivo*.

**Methodology/Principal Findings:**

Eosinophils from blood of healthy donors were treated with AT7519 and apoptosis assessed morphologically and by flow-cytometric detection of annexin-V/propidium iodide staining. AT7519 induced eosinophil apoptosis in a concentration dependent manner. Therapeutic administration of AT7519 in eosinophil-dominant allergic inflammation was investigated using an established ovalbumin-sensitised mouse model of allergic pleurisy. Following ovalbumin challenge AT7519 was administered systemically at the peak of pleural inflammation and inflammatory cell infiltrate, apoptosis and evidence of macrophage phagocytosis of apoptotic eosinophils assessed at appropriate time points. Administration of AT7519 dramatically enhanced the resolution of allergic pleurisy via direct induction of eosinophil apoptosis without detriment to macrophage clearance of these cells. This enhanced resolution of inflammation was shown to be caspase-dependent as the effects of AT7519 were reduced by treatment with a broad spectrum caspase inhibitor (z-vad-fmk).

**Conclusions:**

Our data show that AT7519 induces human eosinophil apoptosis and enhances the resolution of a murine model of allergic pleurisy by inducing caspase-dependent eosinophil apoptosis and enhancing macrophage ingestion of apoptotic eosinophils. These findings demonstrate the utility of cyclin-dependent kinase inhibitors such as AT7519 as potential therapeutic agents for the treatment of eosinophil dominant allergic disorders.

## Introduction

Eosinophils play a key role in the pathogenesis and propagation of allergic diseases, including asthma and allergic rhinitis [1]. In asthmatic patients, eosinophil infiltration into tissue probably contributes to several clinical features, including tissue remodeling and airway hyperresponsiveness [2], [3]. Thus understanding the mechanisms responsible for the recruitment, persistence and clearance of eosinophils in allergic inflammation is required. Increased understanding of the molecular and cellular basis for the action of drugs commonly used in allergic disorders (such as glucocorticosteroids), along with the development of novel treatment strategies, is imperative given that many patient are poorly responsive to glucocorticoid therapy [3], [4].

The accumulation of eosinophils and other leukocytes in tissues depends not only on the number of cells being recruited but also on the number of cells that are cleared from or leave the tissue[5]. In the lung current evidence suggests that transepithelial migration of airway wall leukocytes followed by mucociliary clearance and/or uptake of apoptotic cells by macrophages are important mechanisms responsible for physiological clearance of inflammatory lung cells [6], [7], [8]. Timely apoptosis and subsequent phagocytosis of inflammatory cells ensures that cell membrane integrity is preserved, therefore preventing the release of cytotoxic mediators with subsequent tissue damage and perpetuation of the inflammatory response. Such nonphlogistic clearance of inflammatory eosinophils may be defective in allergic diseases as reduced levels of eosinophil apoptosis in sputum, as well as defective macrophage phagocytosis, are associated with increased asthma severity [9], [10], [11], [12]. Similarly, studies have provided evidence that glucocorticoids, one of the main treatments in severe allergic disorders such as asthma, not only induce eosinophil apoptosis but also enhance macrophage efferocytosis of apoptotic cells[11], [13]. Thus a pharmacological strategy that enhances eosinophil apoptosis and drives subsequent clearance by phagocytes prior to inflammatory cell membrane rupture would make an attractive potential therapeutic agent for the treatment of eosinophil dominant allergic diseases.

One group of agents that has attracted attention is the cyclin-dependent kinase (CDK) inhibitor (CDKi) class of drugs [14], [15], [16], [17]. The CDK enzymes are key regulators of the cell-cycle and are activated by periodic formation of complexes with cyclins (proteins that are present only at specific stages of the cell-cycle) [15], [17]. The activity of CDKs can be modulated by low molecular weight CDKi drugs that attach to the ATP-binding pocket or by modifying the composition of CDK/CDKi drug complexes [18]. Indeed, CDKi drugs are currently undergoing clinical trials for oesophageal, prostate and lung cancers [19]. Furthermore, CDKs have been associated with transcription, neural function and apoptosis [15], [18], [20]. Our group has demonstrated that the CDKi, R-roscovitine, induces neutrophil apoptosis *in vitro* and enhances resolution of established neutrophil-dependent inflammation *in vivo* [17]. In addition, we were the first to show that R-roscovitine induces rapid and efficacious human eosinophil apoptosis [14]. The novel CDKi, AT7519, a selective inhibitor of several CDKs (especially CDKs 1, 2, 4, 5 and 9), has been shown to have antitumor activity *in vitro* and *in vivo* [21], [22], [23], [24]. AT7519 has an attractive biological profile, demonstrating a good aqueous thermodynamic solubility and its synthetic tractability makes it easily amenable to large-scale synthesis. Currently, AT7519 has completed phase-I studies in patients with refractory solid tumours [25]. However, little is known of the effects of CDKi drugs on the molecular mechanisms responsible for eosinophil survival and/or apoptosis *in vivo*.

Here, we investigate the effects of AT7519 on human eosinophil apoptosis *in vitro* as well as the resolution phase of established eosinophilic inflammation *in vivo*. We provide evidence that AT7519 induces a caspase-dependent pro-apoptotic effect in eosinophils and enhances the resolution of established eosinophil-dominant allergic pleurisy by apoptosis of inflammatory cells.

## Results

### The CDKi drug, AT7519, drives primary human eosinophil apoptosis in a concentration-dependent manner

**Figure 1:**
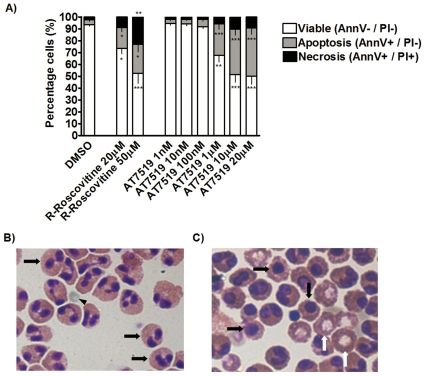
The CDKi drug AT7519 induces apoptosis in primary human eosinophils in a concentration dependent manner. (A) Eosinophils were incubated for 4 h with R-roscovitine (20 µM–50 µM), AT7519 (1 nM–20 µM) or control prior to flow cytometry analysis of AnnV/PI binding to show the percentage of viable, apoptotic and necrotic eosinophils. Data represent mean ± SEM with n = 3. (B-C), Cytocentrifuge images (400xMagnification). (B), Eosinophils after 4 hours of culture; black arrows indicate healthy, viable eosinophils and back arrow head indicating an erythrocyte. (C), Eosinophils after 4 hours of AT7519 treatment (10 µM); black arrows indicate apoptotic eosinophils, white arrows indicate necrotic eosinophils with extrusion of nuclei. *p<0.05, **p<0.01, ***p<0.001 versus DMSO control

We have recently demonstrated that human eosinophils undergo apoptosis following treatment with R-roscovitine *in vitro* [14]. Initial experiments were designed to evaluate whether AT7519 has the same ability to induce eosinophil apoptosis directly *in vitro* as R-roscovitine. This was important to establish as the pharmacological kinase inhibition profile of these agents differs. Human eosinophils were incubated for a 4 h period with increasing concentrations from 1 nM–20 µM AT7519. As a positive control we used increasing concentrations of 20–50 µM R-roscovitine. Apoptosis was assessed by flow cytometric analysis using annexin-V/Propidium iodide (PI) staining. The annexin-V/PI dual negative cells were considered viable, the annexin-V-positive PI-negative cells were considered apoptotic and annexin-V/PI dual positive cells were considered necrotic. AT7519, like R-roscovitine, markedly increased eosinophil apoptosis in a concentration-dependent manner (Figure 1A). However, it is apparent that AT7519 is ∼50 times more potent at inducing apoptosis than R-roscovitine (Figure 1A). It was also observed that at concentrations which induced similar levels of apoptosis (1 µM AT7519 and 50 µM R-Roscovitine) AT7519 was less likely to cause necrosis of eosinophils than R-Roscovitine (Figure 1A). Apoptosis was also assessed morphologically using light microscopy after cytocentrifugation and staining with Diff-Quick™ (Figure 1B-C), confirming flow cytometric data.

To address whether AT7519 induces eosinophil activation, we investigated the effect of the compound alone, and in the presence of eosinophil activating agents on two very sensitive assays of early eosinophil activation; namely i) shape change as measured by increases in forward scatter detected by flow cytometry and ii) intracellular calcium flux as measured by alterations in spectrofluorescence using Fura-2 loaded human eosinophils. AT7519 at 1 µM (a concentration that markedly induces human eosinophil apoptosis) does not induce shape change or a direct increase in intracellular free calcium concentration. Furthermore, the compound does not affect the responses induced by eotaxin, platelet activating factor (PAF) or the formylated chemotactic peptice (fMLP); it neither augments nor, indeed, inhibits the responses to these agonists (data not shown). We are confident that AT7519 does not directly activate eosinophils especially since calcium flux is a key signaling pathway for subsequent eosinophil activation (e.g., LTC_4_ synthesis).

### AT7519 promotes resolution of allergic pleurisy in mice

**Figure 2:**
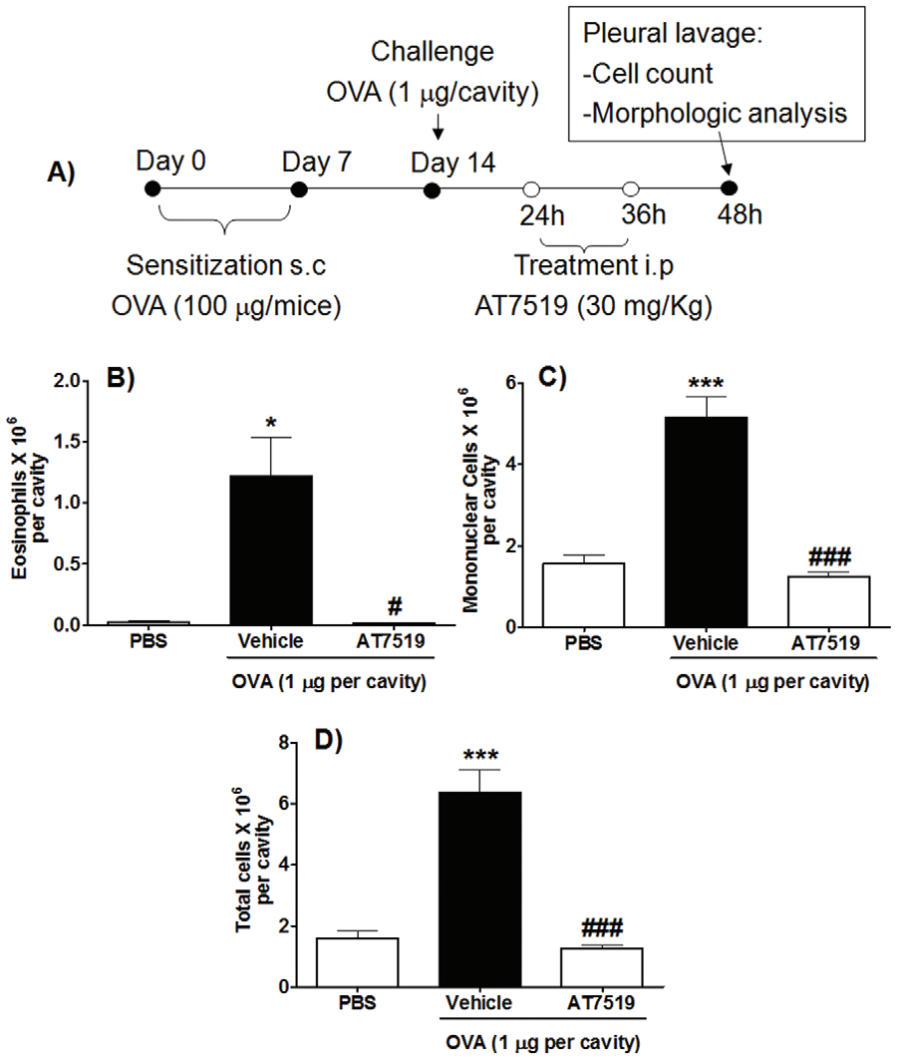
AT7519 promotes resolution of allergic pleurisy *in vivo*. (A) Schematic representation of the protocol of the induction of pleurisy and of the treatment with AT7519. Immunized mice were challenged with OVA or PBS and further 24 h and 36 h later, received a systemic injection of AT7519 or vehicle. The number of eosinophils (B), mononuclear cells (C) and total cell counts (D) were assessed 48 h after antigen-challenge. Results are expressed as mean ± SEM of at least five mice in each group. (B-D) *P<0.05, ***P<0.001 when compared with PBS-injected mice; ^#^P<0.05, ^###^ P<0.001 when compared with vehicle-treated, OVA-injected mice.

Having demonstrated *in vitro* that eosinophil apoptosis was markedly induced by AT7519, we investigated the ability of this agent to resolve eosinophil-dominant inflammation *in vivo*. We used a well-established murine model of acute eosinophilic inflammation, allergic pleurisy [26]. In this model, eosinophil influx is first detectable at 12 h post OVA challenge, becoming maximal at 24–48 h and dropping to near basal levels thereafter. Thus, this experiment evaluated the effects of systemic administration of AT7519 given at the peak of inflammation after the cells have migrated to the cavity (24 h) but before they have been cleared (after 72 h). Pleural lavage was performed 24 h after AT7519 treatment (48 h after OVA-challenge) (See schematic representation in Figure 2A). Injection of 1 µg of ovalbumin (OVA) into the pleural cavity of sensitized mice induced an influx of leukocytes, with an increase in eosinophils, mononuclear cells and total number of leukocytes in OVA-challenged mice (Figures 2B-D). Mice that were treated intraperitoneally *(i.p.)* with AT7519 showed a marked reduction in the numbers of total leucocytes, eosinophils and mononuclear cells in the pleural cavity, consistent with enhanced resolution of established eosinophilic inflammation (Figure 2B-D).

### AT7519 resolves allergic inflammation by driving eosinophil apoptosis and clearance

**Figure 3:**
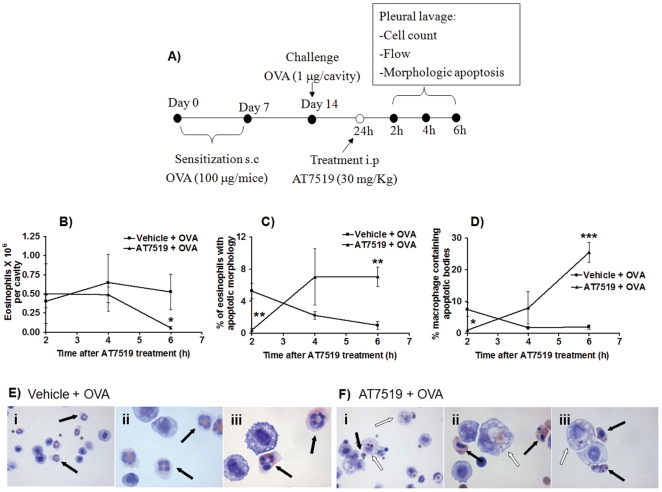
AT7519 drives eosinophil apoptosis and subsequent clearance by macrophages as assessed by morphological analysis. (A) Schematic representation of the experimental protocol. Immunized mice were challenged with OVA and 24 h later received AT7519 or vehicle. Eosinophil number (B), percentage of apoptotic eosinophils (C) and percentage of macrophages containing apoptotic bodies (D) were assed 2, 4 and 6 h after AT7519 treatment. Results are expressed as the mean ± SEM of at least five mice in each group. (B-D) *P<0.05, **P<0.01, ***P<0.001 when compared with vehicle-treated, OVA-injected mice. Representative images from vehicle (E) and AT7519 treated (F) animals are shown (Magnification x400 (i) and x1000 (ii and iii)). In vehicle treated animals (E), black arrows indicate healthy, viable eosinophils while in AT7519-treated animals (F), black arrows indicate typically apoptotic eosinophils and white arrows apoptotic cells inside macrophages.

**Figure 4:**
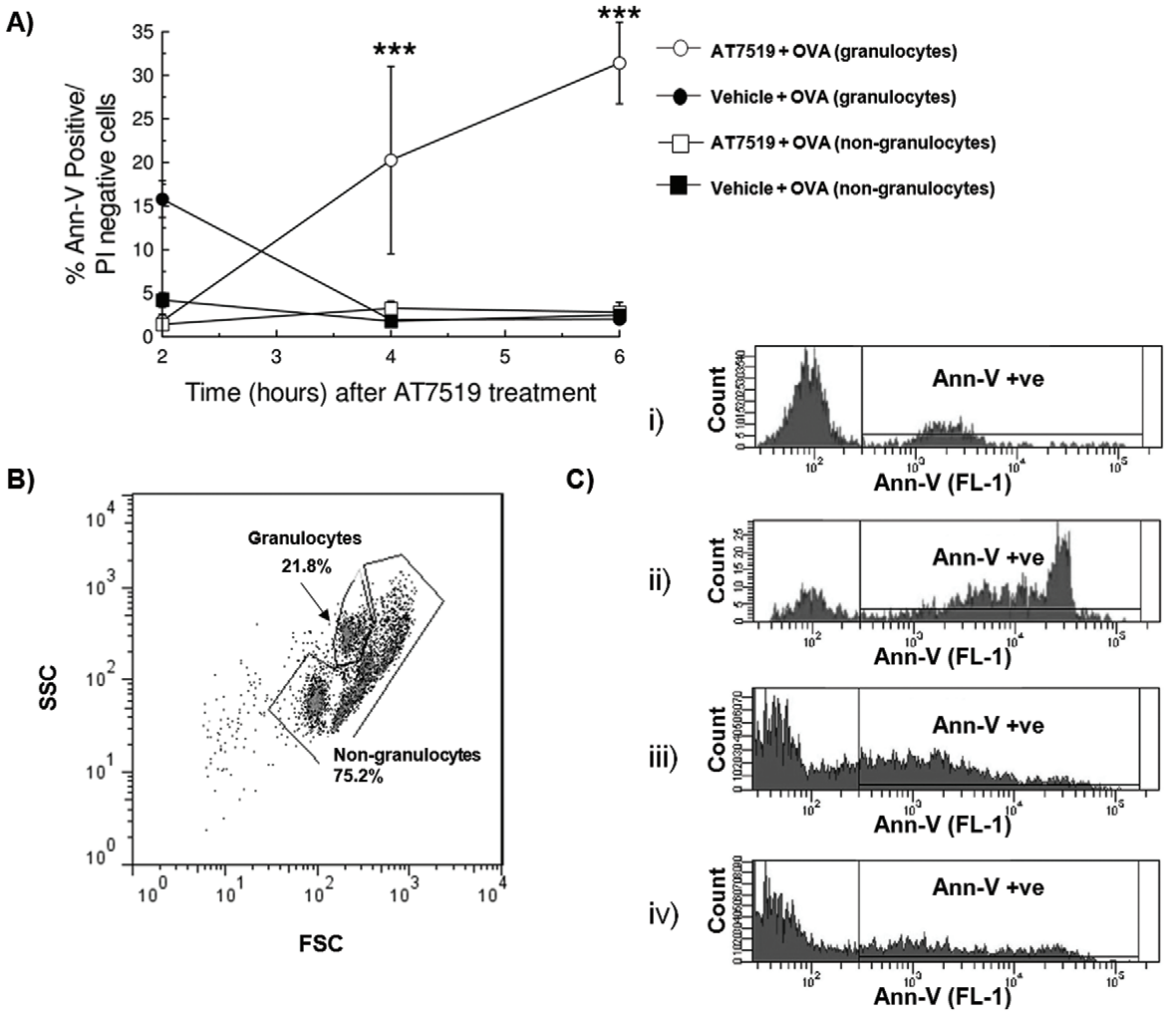
AT7519 drives granulocyte apoptosis as assessed by flow cytometry. Immunized mice were challenged with OVA and 24 h later received AT7519 or vehicle and apoptosis assessed by flow cytometry at 2, 4 and 6 h (A). (B) Typical flow cytometric profile of pleural lavage cells showing granulocytes and non-granulocyte cells gated on the basis of size and granularity. (C) Representative histograms of gated granulocytes from vehicle treated (i) and AT7519 treated (ii) animals as well as representative histograms of gated non-granulocyte cells from vehicle treated (iii) and AT7519 treated (iv) animals at 6 hours post treatment. ***P<0.001 when compared with vehicle-treated, OVA-injected mice.

We next investigated whether the enhanced resolution of allergic pleurisy in the AT7519 treated group was due to induction of eosinophil apoptosis and subsequent clearance of apoptotic cells by macrophages. Given that AT7519 induced rapid eosinophil apoptosis *in vitro* (Figure 1A), earlier time points were chosen for pleural lavage in this set of experiments to ensure that any changes in rates of eosinophil apoptosis were observed (See schematic representation in Figure 3A). In the AT7519 treated group there was a time-dependent decrease of eosinophil number which was mirrored by an increase in the percentage of apoptotic eosinophils as well as the percentage of macrophages containing apoptotic bodies (Figures 3B-D). At 6 h post treatment typical morphology of pleural cavity cells from vehicle treated animals demonstrating viable eosinophils and macrophages without apoptotic bodies (Figure 3E) and AT7519 treated animals demonstrating apoptotic eosinophils as well as apoptotic eosinophils inside macrophages (Figure 3F) are shown. Flow cytometric analysis of annexin-V/PI staining of pleural cells further confirmed the ability of AT7519 to induce time-dependent apoptosis of granulocytes (Figure 4A). A representative flow cytometric profile (forward/side scatter) of pleural lavage cells (Figure 4B) and representative histograms of annexin-V positivity (FL-1, x-axis) of gated granulocytes and non-granulocyte cells are shown for vehicle and AT7519 treated animals (Figure 4C). Importantly AT7519 treatment did not effect rates of apoptosis in non-granulocyte cells (Figure 4A) confirming that enhanced resolution of inflammation was not due to a toxic or apoptosis inducing effect of AT7519 on mononuclear cells *in vivo*.

### AT7519 increases resolution of allergic pleurisy by inducing caspase-dependent apoptosis of inflammatory cells

**Figure 5:**
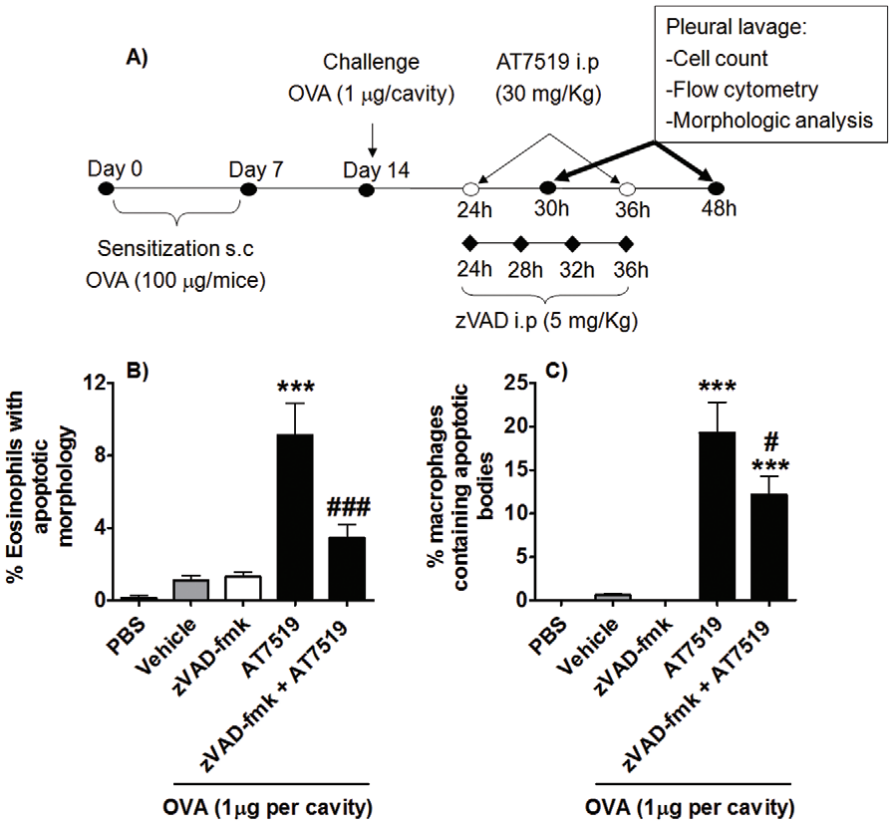
AT7519 effects on eosinophil apoptosis and subsequent clearance by macrophages is caspase dependent. (A) Schematic representation of the experimental protocol. Immunized mice were challenged with OVA and 24 h later were treated with AT7519 and/or zVAD-fmk. The percentage of apoptotic eosinophils (B) and percentage of macrophages containing apoptotic bodies (C) was assessed 30 h after antigen-challenge. Results are expressed as the % cells per cavity, as a mean ± SEM of at least five mice in each group. ***P<0.001 when compared with vehicle-treated, OVA-injected mice. ^#^P<0.05, ^###^ P<0.001 when compared with AT7519-treated, OVA-injected mice.

Having demonstrated enhancement of eosinophil apoptosis by AT7519 *in vivo*, we investigated whether the caspase pathway was involved in the underlying mechanism. To determine this, we utilised a protocol which allows the inhibition of caspase machinery *in vivo* by zVAD-fmk[17]. Immunized animals were treated with AT7519 and/or zVAD-fmk *i.p.* 24 h after antigen-challenge and three additional doses of zVAD-fmk were given (See schematic representation in Figure 5A). The mice were killed 30 h or 48 h post antigen-challenge. We chose the 30 h time point once we observed that the greatest apoptotic response occurred 6 h post AT7519 treatment (24 h OVA+6 h of AT7519, Figures 3C and 4A). Intraperitoneal injection of zVAD-fmk prevented the AT7519-induced increased percentage of apoptotic eosinophils by >62% compared to AT7519-treated animals (Figure 5B) and also reduced the percentage of macrophages containing apoptotic bodies (Figure 5C).

**Figure 6:**
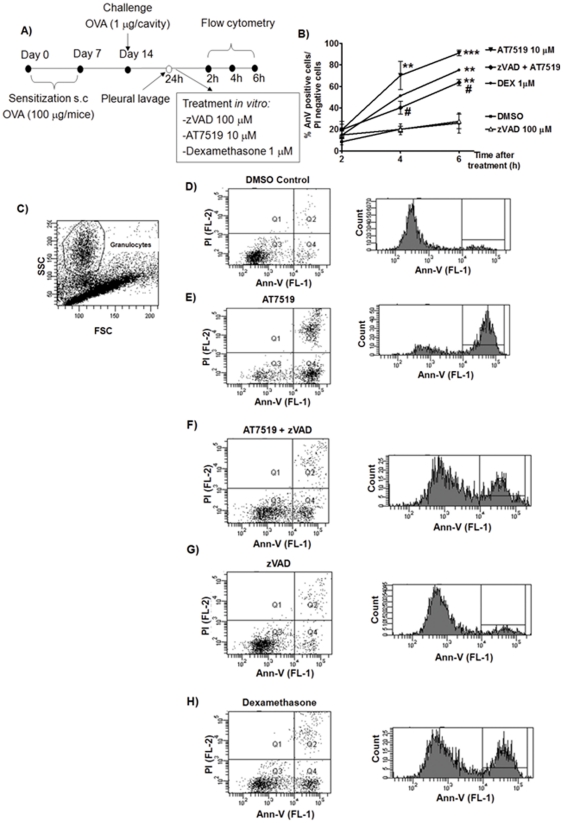
AT7519 induces caspase-dependent apoptosis of inflammatory eosinophils when cultured *ex vivo*. (A) Schematic representation of the experimental protocol. Immunized mice were challenged with OVA and 24 h later cells from the pleural cavity were harvested. Cells were cultured in control (DMSO), zVAD-fmk, AT7519, dexamethasone or combinations for 2, 4 or 6 hours prior to apoptosis assessment by flow cytometric measurement of annexin-V and propidium iodide staining. **P<0.01, ***P<0.001 when compared with DMSO-treated cells; ^#^P<0.05 when compared with zVAD-treated, AT7519-cultered cells. (B) Percentage of apoptotic granulocytes (Ann-V positive/PI negative) shown at each time point. (C-H), Typical flow cytometric profile of pleural lavage cells showing forward/side scatter profile (C) and plots (lower left quadrant AnnV-/PI-, lower right AnnV+/PI- and upper right AnnV+/PI+) and representative annexin-V histograms of gated granulocytes at 4 hours of culture.

The caspase-dependency of the pro-resolution action of AT7519 was further confirmed when inflammatory cells recovered from the pleural cavity of OVA-challenged mice were treated *ex vivo* with AT7519 in combination with zVAD-fmk (see Figure 6A). AT7519 (10 µM) promoted an increased percentage of annexin-V positive/PI negative cells (Figure 6B) when compared to control. When the cells were pre-incubated with zVAD-fmk (100 µM) and then treated with AT7519 30 minutes later, the pro-apoptotic action of AT7519 was blocked (Figure 6B) further corroborating the caspase-dependency of AT7519. As a positive control for induction of eosinophil apoptosis, we used the powerful anti-inflammatory and eosinophil apoptosis–inducing agent, dexamethasone (1 µM)^(30)^. Examples of flow cytometric profiles and representative histograms are shown (Figure 6C-H).

## Discussion

Eosinophils contribute to the pathogenesis of allergic disease [1], [2], [3], [5], and reduced levels of eosinophil apoptosis in sputum correlate with asthma severity [12]. It is important to delineate the mechanisms governing eosinophil lifespan and apoptosis as it is clear that manipulation of eosinophil apoptosis provides an attractive way of physiologically removing the pathological influence of eosinophils in allergic disorders. In addition, phagocytosis of apoptotic cells dampens the inflammatory reaction by switching the ingesting pro-inflammatory macrophages to a more pro-resolution, anti-inflammatory phenotype with enhanced secretion of IL-10 and TGF-β [13], [27], [28], [29], [30].

We have previously shown that the archetypal CDKi, R-roscovitine, induces neutrophil apoptosis *in vitro* by decreasing levels of the pro-survival protein Mcl-1 to override the cyto-protective effects of inflammatory mediators without directly influencing key inflammatory signaling pathways [16]. Importantly, R-roscovitine also enhances resolution of established neutrophil-dependent sterile inflammation *in vivo* using the carrageenan pleurisy model as well as arthritis and bleomycin models [17]. By utilizing transgenic zebrafish Renshaw and colleagues have enabled tracking of all stages of the inflammatory process and have likewise demonstrated the enhanced resolution of inflammation using R-roscovitine [31]. Moreover, Koedel and colleagues [32] have shown that R-roscovitine, in combination with antibiotic therapy, enhances resolution of experimental pneumococcal meningitis in mice by driving neutrophil apoptosis. Recently, we demonstrated that eosinophils undergo apoptosis following treatment with R-roscovitine *in vitro* which is preceded by down-regulation of the anti-apoptotic protein Mcl-1 [14]. Our findings are in accordance with previous studies *in vitro* which show that members of the Bcl-2 family and caspases are central to the mechanism by which eosinophils undergo apoptosis [33], [34].

Here we show that the novel CDKi drug AT7519 induces concentration-dependent eosinophil apoptosis *in vitro* and does so with a potency 50 times greater than R-roscovitive. It is important that any potential pro-resolution agent acting via induction of eosinophil apoptosis is also able to overcome the delay of apoptosis signalled via survival factors present *in vivo*. It is known that the eosinophil apoptosis inducing effects of glucocorticoids are overridden by survival signals conferred from IL-5 [35], perhaps explaining the high frequency of glucocorticoid resistance seen in allergic diseases [4]. R-Roscovitine is able to override the anti-apoptotic effects of IL-5 [36], an effect also observed using AT7519 (data not shown). We specifically selected the already well characterized OVA-induced allergic pleurisy model as we have previously shown that treatment with PI3K inhibitors after antigen challenge markedly reduced eosinophil accumulation, an effect associated with inhibition of Akt phosphorylation and increased apoptosis [26]. Here we show for the first time that a CDKi drug is able to enhance the resolution of established eosinophil-dominant inflammation *in vivo*. Specifically, systemic AT7519 treatment at the peak of the inflammatory process significantly reduced the number of eosinophils, mononuclear cells and total inflammatory cells present in the pleural cavity. Subsequently we demonstrate that AT7519 enhances the resolution of allergic pleurisy by inducing rapid time-dependent eosinophil apoptosis (assessed morphologically and by flow cytometry). Although the absolute levels of apoptosis at any given time point were low compared to the changes observed in total eosinophil number, it is known that small changes in the rates of apoptosis of immune cells can have a significant effect on total cellular populations over time [17], [31], [37], [38]. Apoptotic eosinophils are recognized and ingested as intact cells by macrophages, with macrophages that consume apoptotic granulocytes changing to a pro-resolution phenotype that permits them to release TGF-β and IL-10 [30], [39], [40]. Following AT7519 treatment the percentage of macrophages containing apoptotic bodies in the pleural cavity increased, implying rapid recognition and phagocytosis of apoptotic eosinophils was occurring *in vivo*. Significantly, treatment with AT7519 did not affect rates of apoptosis of non-granulocyte cells recovered from the pleural cavity suggesting that the beneficial effects on inflammatory resolution were not due to a toxic or apoptosis inducing effect on non-granulocyte lineage cells. Therefore reductions in total inflammatory cell and macrophage numbers are most likely a secondary consequence of eosinophil apoptosis, with macrophage numbers returning towards normal levels once the apoptotic cell burden has been fully cleared.

Several studies have demonstrated that zVAD-fmk reduces apoptosis in animal models including sepsis [41] ischemia-reperfusion [42] and bleomycin-induced lung fibrosis [43]. Furthermore, 15-epi-lipoxinA4 overrides myeloperoxidase-driven apoptotic signalling and accelerates the resolution of acute lung injury through a caspase-mediated proapototic effect [44]. Recently we demonstrated that zVAD-fmk prevented rolipram-induced resolution of pleurisy induced by LPS [45]. Similarly, the systemic administration of zVAD-fmk inhibited R-roscovitine-induced decrease in inflammatory cells and oedema formation in the pleural cavity in carrageenan-induced pleural inflammation [17]. Here we have shown that zVAD-fmk treatment markedly decreased the rate of AT7519-induced eosinophil apoptosis as well as the number of macrophages containing apoptotic bodies, demonstrating that AT7519 induces caspase-dependent eosinophil apoptosis *in vivo*. Although zVAD-fmk did not completely abolish the AT7519 mediated apoptotic effect, either *in vivo* or *in vitro*, we feel that this is likely to represent incomplete caspase inhibiton using z-VAD-fmk, rather than the presence of an alternative caspase-independent apoptosis pathway. Such controversy has recently been settled in the neutrophil literature using the newer, more cell permeable and less toxic broad spectrum caspase inhibitor Q-VD-OPh[46], demonstrating that in neutrophils apoptosis can be almost completely inhibited by use of this powerful broad spectrum caspase inhibitor.

Farahi et al. [36] recently reported that R-roscovitine, whilst inducing rapid apoptosis in eosinophils *in vitro*, had little effect on the onset or resolution of eosinophilic inflammation in a murine ovalbumin sensitisation model. Of note, the authors do show a ∼40–50% reduction in eosinophil recovery from bronchoalveolar lavage 72h after the final R-roscovitine challenge, although this was deemed not significant. Furthermore, this group utilised a treatment regimen of 10 mg/kg R-roscovitine delivered *i.p*. Our own *in vivo* work with R-roscovitine [17], as well as several other studies[47], [48] have used a 10-fold higher dose to achieve adequate systemic levels of the drug. This lower dose and/or the well known solubility and dispersion issues with certain CDKi compounds (such as R-Roscovitine) may further explain a lack of any *in vivo* tissue-specific effects observed in the aforementioned study. In addition Farahi et al, like ourselves, have noted that R-roscovitine causes increased eosinophil necrosis in vitro, an effect that is markedly reduced at AT7519 concentrations that induce similar levels of apoptosis. That R-roscovitine may also cause increased eosinophil necrosis *in vivo*, with consequent exacerbation of the inflammatory response, may also explain the relative lack of effect of R-Roscovitine in that model.

In conclusion, our data show that AT7519 induces human eosinophil apoptosis and enhances resolution of allergic pleurisy by inducing caspase-dependent eosinophil apoptosis. Resolution of inflammation is preceded by increased apoptosis and macrophage ingestion of apoptotic eosinophils highlighting the importance of phagocytic clearance of inflammatory cells to the resolution process. We suggest that the non-inflammatory clearance of apoptotic eosinophils by macrophages prevents not only the spillage of histotoxic contents from activated dying cells but may also transform the macrophage to an anti-inflammatory/pro-resolution phenotype with enhanced secretion of TGF-β and IL-10. Based upon our findings, we acknowledge that further studies, ideally using airway eosinophillic-inflammation models and AT7519 as an example of the latest generation of CDKi drugs would be a logical progression. Phenotyping of resolution phase macrophages and measurement of TGF-β and IL-10 *in vivo* would also enhance insight into the mechanisms governing enhanced resolution of inflammation. Local delivery of CDKi drugs directly to the lungs by way of inhaled therapy should be tested for efficacy as a strategy to reduce dose and consequently potential side effects from systemic therapy. We anticipate that our findings will help lead the way to potential therapeutic trials of CDKi drugs in diseases where eosinophils contribute to the pathogenesis and propagation of allergic inflammatory diseases. This may be realised fairly quickly as the CDKi drug used in this study is in the advanced stages of human clinical trials for various cancers and within our own centre, an experimental trial in patients with idiopathic pulmonary fibrosis is under design.

## Materials and Methods

### Ethics Statement

Ethics approval for granulocyte isolation was obtained from the Lothian Research Ethics Committee; approval numbers #08/S1103/38 or #1702/95/4/72, at the University of Edinburgh, Queen's Medical Research Institute, where participants were recruited and experimentation was carried out. Written informed consent was obtained from all participants involved.

Female Balb/C mice (8–10 weeks) were humanely maintained and handled in accordance with the UK Home Office Animals Scientific Procedures Act (Licence Number 60/3829). This licence was approved by the University of Edinburgh Ethical Review Committee (approval ID PL08-08).

### Eosinophil isolation

Granulocytes were isolated from the peripheral venous blood of healthy adult donors by dextran (Pharmacosmos) sedimentation followed by centrifugation through discontinuous PBS-Percoll (GE Healthcare) gradients [49], [50]. Eosinophils were separated from contaminating neutrophils using an immunomagnetic separation step with sheep anti-mouse IgG-Dynabeads (Invitrogen) coated with the murine anti-neutrophil antibody 3G8 as described [51]. Eosinophil purity was routinely greater than 95%.

### Human eosinophil apoptosis assessment

Eosinophils were re-suspended in IMDM (PAA) with 10% FBS (Biosera), penicillin (100 U/mL) and streptomycin (100 U/mL) (PAA). Cells were aliquoted (5×10^6^ cells/mL) into a 96-well-flat-bottomed-flexible-plate (BD Biosciences) in a final volume of 150 µL and incubated with R-roscovitine (Merck), AT7519 (Astex), zVAD-fmk (Bachem), Q-VD-OPh (R&D Systems), IL-5 (R&D Systems) or combinations of these at 37°C with 5% CO_2_ for 4 h. All stock reagents were initially dissolved in dimethylsulphoxide (Sigma) then diluted in buffer yielding a final concentration of 0.2%; a corresponding DMSO control of 0.2% was assessed as an appropriate vehicle control. Apoptosis was assessed by flow cytometry using annexin-V-FLUOS (Roche) in combination with propidium iodide (PI) (Sigma) as described previously [14]. Morphological apoptotic changes were assessed by light microscopy of DiffQuick™ stained cytocentrifuged cells [49], [52].

### Induction of pleurisy

Female Balb/C mice (8–10 weeks) were immunized with ovalbumin (OVA) adsorbed to aluminium hydroxide gel as described previously[26]. Briefly, mice were injected subcutaneously (*s.c.*) on days 1 and 7 with 0.2 mL of a solution containing 100 µg of OVA and 70 µg of aluminium hydroxide. Sensitized mice were then challenged with OVA (1 µg/cavity, in a total volume of 100 µl intrapleurally) or PBS and a further 24 h and 36 h later, received systemic AT7519 (30 mg/kg, intraperitoneally (*i.p.*)) or PBS vehicle. The cells present in the pleural cavity were harvested at different times after antigen-challenge by washing the cavity with 2 mL of PBS and total cell counts performed in a NucleoCounter® system using NucleoCassette™ (Chemometec, Denmark). For the experiments evaluating leukocyte apoptosis, infiltrating leukocytes were examined at 2, 4 and 6 h (flow cytometry) and 30 and 48 h (morphologic apoptosis) after drug treatment. Differential cell counts were performed on cytocentrifugation preparations stained with DiffQuick™. The results are presented as the number or % cells per cavity as indicated in figures.

### Assessment of leukocyte apoptosis in pleurisy model

Apoptosis was assessed as described previously[17]. Briefly, cells (5×10^4^) collected 30 or 48 h after antigen-challenge were cytocentrifuged, fixed, stained and counted using oil immersion microscopy (x100 objective) to determine the proportion of cells with distinctive apoptotic morphology, with five hundred cells counted per slide. Assessment of apoptosis was also performed by flow cytometry using annexin-V-FLUOS in combination with propidium iodide (PI). Annexin-V was diluted 1\500 in binding buffer (HBSS+Ca^2+^) and 280 µL added to 20 µL of cells (5×10^6^ cells\mL). Samples were then incubated on ice at 4°C for 10minutes. Immediately prior to processing, 1 µL PI (1 mg\mL) per sample was added. Results are expressed as cells undergoing the early stage of apoptosis quantified by staining with annexin-V but not PI. The cells were selected based on size and granularity, allowing separate analysis of granulocyte population.

### Administration of zVAD-fmk

Twenty-four hours after intrapleural injection of OVA, mice were injected *i.p* with 30 mg/kg of AT7519 and/or 5 mg/kg of zVAD-fmk. Three additional doses of zVAD-fmk were given *i.p* 4, 8, 12 h later and mice were killed 30 h after the OVA challenge.

### Ex vivo culture of leukocytes

The cells present in the pleural cavity from mice immunized according to the protocol above were harvested at 24 h after antigen-challenge by washing the cavity with 2 mL of PBS and were aliquoted (5×10^6^ cells/mL) into a 96-well-flat-bottomed-flexible-plate (BD Biosciences) in a final volume of 150 µL at 37°C with 5% CO_2_ for 2, 4 and 6 h.

### Statistical analysis

All *in vitro* experiments were performed at least three times with each experiment carried out in triplicate. All *in vivo* experiments included 6 mice per group. Data were expressed as the mean±SEM. The principal statistical test used for comparison of multiple groups was one-way ANOVA with post hoc multivariate analysis by Student Newman-Keuls (with a 95% confidence interval). In order to test statistical significance between two groups we used unpaired Student's t test. Differences were considered significant at *p<0.05, **p<0.01, ***p<0.001.

## References

[1] Rothenberg ME, Hogan SP (2006). The eosinophil.. Annu Rev Immunol.

[2] Venge P (2010). The eosinophil and airway remodelling in asthma.. Clin Respir J.

[3] Kay AB (2005). The role of eosinophils in the pathogenesis of asthma.. Trends Mol Med.

[4] Barnes PJ, Adcock IM (2009). Glucocorticoid resistance in inflammatory diseases.. Lancet.

[5] Bradley BL, Azzawi M, Jacobson M, Assoufi B, Collins JV (1991). Eosinophils, T-lymphocytes, mast cells, neutrophils, and macrophages in bronchial biopsy specimens from atopic subjects with asthma: comparison with biopsy specimens from atopic subjects without asthma and normal control subjects and relationship to bronchial hyperresponsiveness.. J Allergy Clin Immunol.

[6] Persson C, Uller L (2010). Transepithelial exit of leucocytes: inflicting, reflecting or resolving airway inflammation?. Thorax.

[7] Leitch AE, Duffin R, Haslett C, Rossi AG (2008). Relevance of granulocyte apoptosis to resolution of inflammation at the respiratory mucosa.. Mucosal Immunol.

[8] Haslett C (1999). Granulocyte apoptosis and its role in the resolution and control of lung inflammation.. Am J Respir Crit Care Med.

[9] Hamid Q, Tulic M (2009). Immunobiology of asthma.. Annu Rev Physiol.

[10] Fitzpatrick AM, Holguin F, Teague WG, Brown LA (2008). Alveolar macrophage phagocytosis is impaired in children with poorly controlled asthma.. J Allergy Clin Immunol.

[11] Woolley KL, Gibson PG, Carty K, Wilson AJ, Twaddell SH (1996). Eosinophil apoptosis and the resolution of airway inflammation in asthma.. Am J Respir Crit Care Med.

[12] Duncan CJ, Lawrie A, Blaylock MG, Douglas JG, Walsh GM (2003). Reduced eosinophil apoptosis in induced sputum correlates with asthma severity.. Eur Respir J.

[13] Michlewska S, Dransfield I, Megson IL, Rossi AG (2009). Macrophage phagocytosis of apoptotic neutrophils is critically regulated by the opposing actions of pro-inflammatory and anti-inflammatory agents: key role for TNF-alpha.. FASEB J.

[14] Duffin R, Leitch AE, Sheldrake TA, Hallett JM, Meyer C (2009). The CDK inhibitor, R-roscovitine, promotes eosinophil apoptosis by down-regulation of Mcl-1.. FEBS Lett.

[15] Leitch AE, Haslett C, Rossi AG (2009). Cyclin-dependent kinase inhibitor drugs as potential novel anti-inflammatory and pro-resolution agents.. Br J Pharmacol.

[16] Leitch AE, Riley NA, Sheldrake TA, Festa M, Fox S (2010). The cyclin-dependent kinase inhibitor R-roscovitine down-regulates Mcl-1 to override pro-inflammatory signalling and drive neutrophil apoptosis.. Eur J Immunol.

[17] Rossi AG, Sawatzky DA, Walker A, Ward C, Sheldrake TA (2006). Cyclin-dependent kinase inhibitors enhance the resolution of inflammation by promoting inflammatory cell apoptosis.. Nat Med.

[18] Knockaert M, Greengard P, Meijer L (2002). Pharmacological inhibitors of cyclin-dependent kinases.. Trends Pharmacol Sci.

[19] Senderowicz AM (2003). Novel small molecule cyclin-dependent kinases modulators in human clinical trials.. Cancer Biol Ther.

[20] Menn B, Bach S, Blevins TL, Campbell M, Meijer L (2010). Delayed treatment with systemic (S)-roscovitine provides neuroprotection and inhibits in vivo CDK5 activity increase in animal stroke models.. PLoS One.

[21] Wyatt PG, Woodhead AJ, Berdini V, Boulstridge JA, Carr MG (2008). Identification of N-(4-piperidinyl)-4-(2,6-dichlorobenzoylamino)-1H-pyrazole-3-carboxamide (AT7519), a novel cyclin dependent kinase inhibitor using fragment-based X-ray crystallography and structure based drug design.. J Med Chem.

[22] Squires MS, Feltell RE, Wallis NG, Lewis EJ, Smith DM (2009). Biological characterization of AT7519, a small-molecule inhibitor of cyclin-dependent kinases, in human tumor cell lines.. Mol Cancer Ther.

[23] Squires MS, Cooke L, Lock V, Qi W, Lewis EJ (2010). AT7519, a cyclin-dependent kinase inhibitor, exerts its effects by transcriptional inhibition in leukemia cell lines and patient samples.. Mol Cancer Ther.

[24] Santo L, Vallet S, Hideshima T, Cirstea D, Ikeda H (2010). AT7519, A novel small molecule multi-cyclin-dependent kinase inhibitor, induces apoptosis in multiple myeloma via GSK-3beta activation and RNA polymerase II inhibition.. Oncogene.

[25] Mahadevan D, Plummer R, Squires MS, Rensvold D, Kurtin S (2011). A phase I pharmacokinetic and pharmacodynamic study of AT7519, a cyclin-dependent kinase inhibitor in patients with refractory solid tumors.. Ann Oncol 2011.

[26] Pinho V, Souza DG, Barsante MM, Hamer FP, De Freitas MS (2005). Phosphoinositide-3 kinases critically regulate the recruitment and survival of eosinophils in vivo: importance for the resolution of allergic inflammation.. J Leukoc Biol.

[27] Fadok VA, Bratton DL, Konowal A, Freed PW, Westcott JY (1998). Macrophages that have ingested apoptotic cells in vitro inhibit proinflammatory cytokine production through autocrine/paracrine mechanisms involving TGF-beta, PGE2, and PAF.. J Clin Invest.

[28] Duffin R, Leitch AE, Fox S, Haslett C, Rossi AG (2010). Targeting granulocyte apoptosis: mechanisms, models, and therapies.. Immunol Rev.

[29] Serhan CN, Savill J (2005). Resolution of inflammation: the beginning programs the end.. Nat Immunol.

[30] Gilroy DW, Lawrence T, Perretti M, Rossi AG (2004). Inflammatory resolution: new opportunities for drug discovery.. Nat Rev Drug Discov.

[31] Loynes CA, Martin JS, Robertson A, Trushell DM, Ingham PW (2010). Pivotal Advance: Pharmacological manipulation of inflammation resolution during spontaneously resolving tissue neutrophilia in the zebrafish.. J Leukoc Biol.

[32] Koedel U, Frankenberg T, Kirschnek S, Obermaier B, Hacker H (2009). Apoptosis is essential for neutrophil functional shutdown and determines tissue damage in experimental pneumococcal meningitis.. PLoS Pathog.

[33] Hallett JM, Leitch AE, Riley NA, Duffin R, Haslett C (2008). Novel pharmacological strategies for driving inflammatory cell apoptosis and enhancing the resolution of inflammation.. Trends Pharmacol Sci.

[34] Simon HU (2001). Regulation of eosinophil and neutrophil apoptosis--similarities and differences.. Immunol Rev.

[35] Brode S, Farahi N, Cowburn AS, Juss JK, Condliffe AM (2010). Interleukin-5 inhibits glucocorticoid-mediated apoptosis in human eosinophils.. Thorax.

[36] Farahi N, Uller L, Juss JK, Langton AJ, Cowburn AS (2011). Effects of the cyclin-dependent kinase inhibitor R-roscovitine on eosinophil survival and clearance.. Clin Exp Allergy.

[37] Brazil TJ, Dagleish MP, McGorum BC, Dixon PM, Haslett C (2005). Kinetics of pulmonary neutrophil recruitment and clearance in a natural and spontaneously resolving model of airway inflammation.. Clin Exp Allergy.

[38] Negredo E, Massanella M, Puig J, Perez-Alvarez N, Gallego-Escuredo JM (2010). Nadir CD4 T cell count as predictor and high CD4 T cell intrinsic apoptosis as final mechanism of poor CD4 T cell recovery in virologically suppressed HIV-infected patients: clinical implications.. Clin Infect Dis.

[39] Voll RE, Herrmann M, Roth EA, Stach C, Kalden JR (1997). Immunosuppressive effects of apoptotic cells.. Nature.

[40] Huynh ML, Fadok VA, Henson PM (2002). Phosphatidylserine-dependent ingestion of apoptotic cells promotes TGF-beta1 secretion and the resolution of inflammation.. J Clin Invest.

[41] Qin S, Wang H, Yuan R, Li H, Ochani M (2006). Role of HMGB1 in apoptosis-mediated sepsis lethality.. J Exp Med.

[42] Mersmann J, Zacharowski PA, Schmitz I, Zacharowski K (2008). Caspase inhibitor zVAD.fmk reduces infarct size after myocardial ischaemia and reperfusion in rats but not in mice.. Resuscitation.

[43] Kuwano K, Kunitake R, Maeyama T, Hagimoto N, Kawasaki M (2001). Attenuation of bleomycin-induced pneumopathy in mice by a caspase inhibitor.. Am J Physiol Lung Cell Mol Physiol.

[44] El Kebir D, Jozsef L, Pan W, Wang L, Petasis NA (2009). 15-epi-lipoxin A4 inhibits myeloperoxidase signaling and enhances resolution of acute lung injury.. Am J Respir Crit Care Med.

[45] Sousa LP, Lopes F, Silva DM, Tavares LP, Vieira AT (2010). PDE4 inhibition drives resolution of neutrophilic inflammation by inducing apoptosis in a PKA-PI3K/Akt-dependent and NF-kappaB-independent manner.. J Leukoc Biol.

[46] Wardle DJ, Burgon J, Sabroe I, Bingle CD, Whyte MK (2011). Effective caspase inhibition blocks neutrophil apoptosis and reveals Mcl-1 as both a regulator and a target of neutrophil caspase activation.. PLoS One.

[47] McClue SJ, Blake D, Clarke R, Cowan A, Cummings L (2002). In vitro and in vivo antitumor properties of the cyclin dependent kinase inhibitor CYC202 (R-roscovitine).. Int J Cancer.

[48] Liebl J, Weitensteiner SB, Vereb G, Takacs L, Furst R (2010). Cyclin-dependent kinase 5 regulates endothelial cell migration and angiogenesis.. J Biol Chem.

[49] Ward C, Chilvers ER, Lawson MF, Pryde JG, Fujihara S (1999). NF-kappaB activation is a critical regulator of human granulocyte apoptosis in vitro.. J Biol Chem.

[50] Haslett C, Guthrie LA, Kopaniak MM, Johnston RB, Henson PM (1985). Modulation of multiple neutrophil functions by preparative methods or trace concentrations of bacterial lipopolysaccharide.. Am J Pathol.

[51] Rossi AG, Haslett C, Hirani N, Greening AP, Rahman I (1998). Human circulating eosinophils secrete macrophage migration inhibitory factor (MIF). Potential role in asthma.. J Clin Invest.

[52] Fujihara S, Ward C, Dransfield I, Hay RT, Uings IJ (2002). Inhibition of nuclear factor-kappaB activation un-masks the ability of TNF-alpha to induce human eosinophil apoptosis.. Eur J Immunol.

